# Prevalence and Risk Factors of Ovarian Metastases in Breast Cancer Patients < 41 Years of Age in the Netherlands: A Nationwide Retrospective Cohort Study

**DOI:** 10.1371/journal.pone.0168277

**Published:** 2017-01-26

**Authors:** Inge T. A. Peters, Erik W. van Zwet, Vincent T. H. B. M. Smit, Gerrit Jan Liefers, Peter J. K. Kuppen, Carina G. J. M. Hilders, J. Baptist Trimbos

**Affiliations:** 1 Department of Gynecology, Leiden University Medical Center, Leiden, the Netherlands; 2 Department of Medical Statistics, Leiden University Medical Center, Leiden, the Netherlands; 3 Department of Pathology, Leiden University Medical Center, Leiden, the Netherlands; 4 Department of Surgery, Leiden University Medical Center, Leiden, the Netherlands; 5 Department of Gynecology, Reinier de Graaf Hospital, Delft, the Netherlands; University of South Alabama Mitchell Cancer Institute, UNITED STATES

## Abstract

**Purpose:**

Breast cancer is one of the primary indications for cryopreservation and subsequent autotransplantation of ovarian tissue. The safety of this fertility preservation method remains questionable, as the presence of disseminated breast tumor cells cannot yet be excluded in the ovarian autografts. We explored the prevalence of ovarian metastases among young breast cancer patients and determined risk factors for the development of ovarian metastases.

**Methods:**

Using the nationwide database of the Dutch Pathology Registry (PALGA), we identified a cohort of 2648 women with primary invasive breast cancer at age < 41 years in the period 2000–2010 in the Netherlands who subsequently underwent an oophorectomy. From this source population, all cases who had histologically confirmed ovarian metastases were included. For each case of whom clinical data were available, one control without ovarian metastases who matched the time interval between breast cancer diagnosis and oophorectomy was selected. Data were collected on patient characteristics, diagnosis, treatment and follow-up.

**Results:**

Ovarian metastases were found in 63 out of 2648 patients who met the inclusion criteria. The risk of developing ovarian metastases increased with time passed since breast cancer diagnosis. Multivariate logistic regression analyses showed significant association between tumor stage and the development of ovarian metastases (*p* = 0.024).

**Conclusions:**

The prevalence of ovarian metastases was 2.4% among young breast cancer patients. Early ovary removal may reduce the risk of developing ovarian metastases. In breast cancer patients with tumors > 5 cm and/or inflammatory carcinoma, we recommend a cautious approach to ovarian tissue autotransplantation.

## Introduction

Breast cancer is the most frequently diagnosed malignancy among women with worldwide around 230.000 new cases in 2015 [1]. Approximately 5% of these women were aged younger than 40 years at the time of diagnosis [2]. In these young women, chemotherapy may result in premature ovarian failure [3] and could pose a threat to ovarian function and future childbearing potential. Fertility preservation is therefore of crucial importance. In addition to cryopreservation of embryos and oocytes, which are currently the most established options to preserve fertility, cryopreservation followed by autotransplantation of ovarian tissue is progressively emerging. This approach does not only offer young women the chance to conceive and have their own genetic offspring, but also provides the opportunity to restore their endocrine function [4,5]. In recent series, restoration of ovarian activity has been observed in 93% of cases [6] and 60 live births have now been reported [7].

Despite these favorable outcomes, the safety of this method remains of great concern, since ovarian tissue may contain malignant cells derived from the primary invasive breast tumor. Previous studies, mainly comprising autopsies, prophylactic and therapeutic oophorectomies, showed that ovarian metastases occur in 13–47% of breast cancer patients [8–10]. By contrast, in early-stage breast cancer patients who were eligible for cryopreservation of ovarian tissue, immunohistochemical examination of cortical ovarian biopsies did not disclose any malignant cells [11–13]. Quantitative PCR analysis of frozen-thawed cortical ovarian fragments from patients with advanced-stage breast cancer revealed cells that expressed the mammaglobin B (*MGB2*) gene, which is associated with breast cancer [14]. However, whether these cells bear any malignant potential remains unclear.

Although the results with respect to cryopreservation of ovarian tissue are relatively reassuring, it should be stressed that only a few cortical ovarian fragments were included for analysis, since the current tumor detection methods (i.e. immunohistochemistry, PCR analysis) render the ovarian tissues unsuitable for autotransplantation. It therefore remains difficult to estimate the prevalence of ovarian metastases in breast cancer patients who are considered for ovarian tissue cryopreservation. Furthermore, as a consequence of this approach, malignant cells that have disseminated to the ovarian autografts cannot be excluded and might be reimplanted upon autotransplantation of ovarian tissue.

In this study, we aimed to explore the prevalence of ovarian metastases among young patients diagnosed with primary invasive breast cancer in order to assess the risk of reimplanting malignant cells following autotransplantation of ovarian tissue. In addition, we identified risk factors associated with the presence of ovarian metastases in young patients diagnosed with primary invasive breast cancer in order to more thoroughly define selection criteria for cryopreservation of ovarian tissue in breast cancer patients.

## Material and Methods

### Patient selection and data collection of the study population

**Fig 1:**
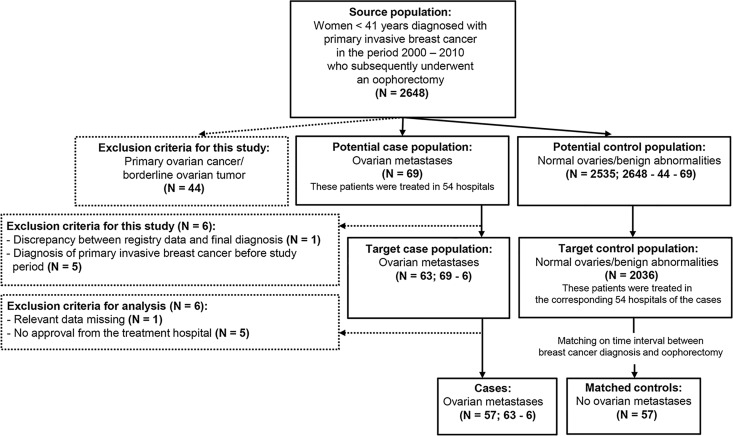
Flow chart of selection of cases and matched controls. The source population was compiled by the Dutch histopathology and cytopathology network. The exclusion criteria are indicated in the dotted boxes.

Via a nationwide search performed by PALGA, the Dutch histopathology and cytopathology network and archive that encompasses all pathology laboratories within the Netherlands [15], a source population was compiled. This source population consisted of all patients who were diagnosed with primary invasive breast cancer at age < 41 years in the period 2000–2010 who subsequently underwent a unilateral or bilateral oophorectomy for any reason (n = 2648; Fig 1). From this source population, all patients who had histologically confirmed ovarian metastases derived from primary invasive breast cancer were selected (n = 69; cases). Patients who were diagnosed with primary ovarian cancer or a borderline ovarian malignancy were excluded (n = 44). From the remaining group of patients who had normal ovaries or benign ovarian abnormalities (n = 2535; controls), all patients who were treated in the same hospitals as the cases were taken (n = 2036). For each case of whom clinical data were available (n = 57), one control without ovarian metastases was included who matched the time interval between the diagnosis of breast cancer and oophorectomy (n = 57; matched controls). Clinical data were extracted from the patient’s files after approval by the medical ethical committee of the Leiden University Medical Center (protocol number P14.106) and the local ethical committee of the participating hospitals. Data were collected on patient characteristics, diagnosis of breast cancer, treatment and follow-up. Furthermore, data were sought on date of oophorectomy, age at oophorectomy, reasons to perform ovarian surgery and diagnosis. From the primary invasive breast tumors in which the *HER2/neu* gene amplification status was not yet determined, formalin-fixed paraffin-embedded (FFPE) tissue samples were requested from the pathology laboratories. Following this, immunohistochemistry was performed on 3-μm thick FFPE tissue sections using primary antibodies against Her2/neu (ERBB2, rabbit polyclonal, Dako, Denmark), as described previously [16]. Primary invasive breast tumors that showed immunohistochemical reactions of 0 and 1+ were considered negative. In primary invasive breast tumors that showed 2+ or 3+ immunohistochemical reaction [17], chromogenic silver in situ hybridization (SISH) was carried out using the Ventana SISH kit on Benchmark XT to establish the final *HER2/neu* status (amplification or no amplification) [18]. All patient samples and clinical data were handled in accordance with the medical ethics guidelines described in the Code of Conduct for Proper Secondary Use of Human Tissue of the Dutch Federation of Biomedical Scientific Societies (FMWV) [19].

### Validation of the control population

**Fig 2:**
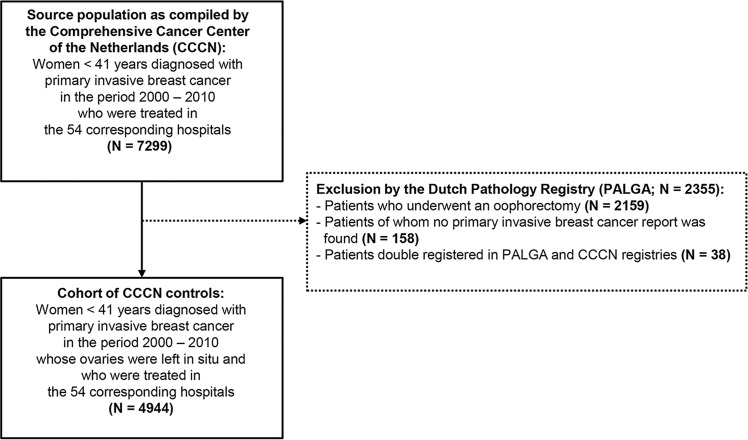
Flow chart of selection of patients diagnosed with primary invasive breast cancer at age < 41 years who did not undergo an oophorectomy. This cohort of patients was compiled by the Comprehensive Cancer Center the Netherlands (CCCN) and indicated as CCCN controls in the study. The exclusion criteria are indicated in the dotted box.

In order to estimate whether the matched controls to some extent also reflected women diagnosed with primary invasive breast cancer at age < 41 years whose ovaries remained *in situ*, the matched controls were compared to a cohort of patients who did not undergo an oophorectomy. To this end, all patients diagnosed with primary invasive breast cancer at age < 41 years who were treated in the corresponding 54 hospitals, were selected from the Dutch Cancer Registry (n = 7299; Fig 2). After notification by PALGA, patients who had undergone an oophorectomy or who were either not or double registered in the PALGA registry, were excluded (n = 2355). The remaining group of patients exclusively consisted of breast cancer patients who were younger than 41 years of age at the time of diagnosis and did not undergo ovarian surgery (n = 4944). From these patients, data on the diagnosis of breast cancer, staging and treatment were collected from the medical records by trained registry personnel using the registration and coding manual of the Comprehensive Cancer Center the Netherlands (CCCN). This group of patients was further indicated as CCCN controls in this study.

### Statistical analysis

Statistical analysis was performed using SPSS version 23.0 (IBM, Armonk, NY). Logistic regression analyses were used to identify predictors for the development of ovarian metastases in the study population and for comparing the current control group with the cohort of CCCN controls. Missing values were accounted for by 10-fold multiple imputation, in which all risk factors and the case-control status in the imputation models were included. In some cases, logistic regression analyses could not be performed because of empty categories. In those cases, the Pearson Chi-square test was used. Factors that were associated with the development of ovarian metastases (*p* < 0.100) in univariate logistic regression models were included in multivariate logistic regression analyses. Survival rates were calculated according to the Kaplan Meier method. Statistical significance was assigned at the level of *p* < 0.05.

## Results

### Prevalence of ovarian metastases

According to the PALGA registry, 2648 patients were diagnosed with primary invasive breast cancer at age < 41 years in the period 2000–2010 who subsequently underwent a unilateral or bilateral oophorectomy (Fig 1). Among these women, 69 patients (2.6%) had histologically confirmed ovarian metastases. Yet, in one patient the registry data did not correspond to the final pathological diagnosis. Moreover, in five patients the diagnosis of primary invasive breast cancer was made before the study period. Thus, strictly, ovarian metastases were found with a prevalence of 2.4% (63 out of 2642) in patients with primary invasive breast cancer at age < 41 years in the period 2000–2010 in the Netherlands.

### Clinicopathological characteristics of the cases

Clinical data were available for 57 patients diagnosed with primary invasive breast cancer and ovarian metastases (Fig 1). The median age at the time of breast cancer diagnosis was 37.0 years (range 28–40 years). Ten patients (17.5%) were tested for the presence of a *BRCA* gene mutation and one of them resulted positive; the *BRCA* gene mutation status in the remaining patients was unknown. Forty-four patients (77.2%) were diagnosed with invasive ductal breast cancer and eight patients (14.0%) were diagnosed with invasive lobular breast cancer. The remaining five patients (8.8%) had invasive ductolobular breast cancer. Fifty-one patients (89.5%) had hormone-sensitive breast cancer. *HER-2/neu* gene amplification was observed in eight of 56 tumor samples tested (14.3%); of the remaining tumor, no tissue was available. The majority of patients had positive axillary lymph nodes and 41 patients (71.9%) had tumors larger than 2 cm in diameter of whom five patients presented with inflammatory breast cancer. Nine patients (15.8%) had distant metastases outside the ovary at the time of diagnosis of primary invasive breast cancer; eight patients had bone metastases of whom two had synchronous liver metastases, and one patient was diagnosed with both pulmonary and retinal metastases. Surgical resection of the primary breast tumor was performed by either breast conserving surgery (18 patients; 31.6%) or mastectomy (33 patients; 57.9%). Six patients (10.5%) did not undergo any surgical treatment, because of diffuse metastatic disease. Adjuvant chemotherapy was administered to 35 patients (61.4%), 36 patients (63.2%) underwent locoregional radiotherapy and 45 patients (78.9%) received hormonal treatment.

The median time between the diagnosis of breast cancer and oophorectomy was 48.7 months (range 0.3–141.8 months). Apart from the nine patients who already had distant metastases at the onset of breast cancer, 33 patients (57.9%) developed a locoregional or distant recurrence prior to oophorectomy. The presence of ovarian metastases was the first manifestation of recurrent disease in fifteen patients (26.3%). Thirty-four patients (59.6%) had ovarian metastases in both ovaries. In seven patients (12.3%) one or both Fallopian tubes were involved, whereas in 33 patients (57.9%) the Fallopian tubes were free of metastatic disease. Of the remaining 17 patients (29.8%), no data on the Fallopian tubes were available. Seven patients (12.3%) had peritonitis carcinomatosa at the time of oophorectomy. The median duration of follow-up was 152.8 months (range 9.9–166.6 months). During follow-up, 43 patients (75.4%) died, all because of metastatic breast cancer. The median time from the diagnosis of ovarian metastases to death was 24.0 months (range 2.3–118.7 months). The 5-year disease-specific survival was 69.5%.

### Risk factor analysis for the development of ovarian metastases

The time interval between the diagnosis of breast cancer and oophorectomy significantly differed between the 63 cases who were diagnosed with ovarian metastases and who met the inclusion criteria, and the 2535 controls without ovarian metastases in the source population, 47.0 and 32.0 months, respectively (*p* = 0.002). In order to identify baseline risk factors that are associated with the development of ovarian metastases, the time interval between the diagnosis of breast cancer and oophorectomy should be comparable between the cases and controls. Therefore, the 57 cases of whom clinical data were available, were matched on this time interval to an equally large cohort of controls (Fig 1). Table 1 shows the indications for oophorectomy in the cases and the matched controls. The cases had significantly more often abnormal ovaries on preoperative transvaginal ultrasonography or MRI than the matched controls, 26.3% versus 3.5%, respectively (*p* = 0.000). The two matched controls who presented with abnormal ovaries were diagnosed with a serous cystadenoma and an epithelioid cell granuloma, respectively. The 42 cases who presented with normal ovaries on transvaginal ultrasound underwent oophorectomy because of prophylactic or therapeutic reasons. In those cases, the ovarian metastases were clinically indolent. This emphasizes the need to determine which young breast cancer patient is at risk of developing ovarian metastases.

**Table 1:** Indications for oophorectomy in patients diagnosed with primary invasive breast cancer at age < 41 years with and without ovarian metastases.

	Cases		Matched controls		*p*-value
	N = 57	%	N = 57	%	
Indication for oophorectomy					0.000
Prophylactic because of breast cancer	11	19.3	39	68.4	
Therapeutic because of breast cancer	31	54.4	15	26.3	
Abnormal ovaries on ultrasound	15	26.3	2	3.5	
Unknown	0	0.0	1	1.8	

The cases with ovarian metastases were matched on the time interval between the diagnosis of breast cancer and oophorectomy to an equally large cohort of controls without ovarian metastases, as shown in the flow chart of Fig 1. The Pearson Chi-square test was used to compare the indications for oophorectomy between the cases and matched controls. *P*-values < 0.05 were considered statistically significant.

Table 2 shows the results of the univariate and multivariate logistic regression analyses that were performed in the matched case-control population. Univariate logistic regression analyses revealed that the risk of developing ovarian metastases significantly increased with tumor size and the presence of inflammatory breast cancer, the number of positive lymph nodes and the presence of distant metastases. In the multivariate logistic regression analyses, only a larger tumor size (i.e. > 5 cm) and the presence of inflammatory breast cancer was significantly associated with the development of ovarian metastases (*p* = 0.024). The presence of distant metastases could not be included in the multivariate logistic regression analyses, as none of the matched controls had clinical evidence of distant metastases at the time of breast cancer diagnosis.

**Table 2:** Clinicopathological characteristics of patients diagnosed with primary invasive breast cancer at age < 41 years with and without ovarian metastases.

Characteristics	Cases		Matched controls		Univariate analysis	Multivariate analysis
	N = 57	%	N = 57	%	*p*-value	*p*-value
Age at diagnosis of breast cancer, years—median (range)	37	(28–40)	36	(27–40)	0.730	n.a.
Breast tumor localization					0.271	n.a.
• Left • Right • Both	30	52.6	27	47.4		
	25	43.9	30	52.6		
	2	3.5	0	0.0		
Histological subtype					0.333	n.a.
• Ductal • Lobular • Ductolobular	44	77.2	52	91.2		
	8	14.0	4	7.0		
	5	8.8	1	1.8		
Scarff-Bloom-Richardson grade					0.174	n.a.
• I • II • III • Missing	5	8.8	5	8.8		
	30	52.6	20	35.1		
	22	38.6	31	54.4		
	0	0.0	1	1.8		
Estrogen receptor					0.055	0.084
• Negative • Positive	6	10.5	14	24.6		
	51	89.5	43	75.4		
Progesterone receptor					0.167	n.a.
• Negative • Positive • Missing	10	17.5	17	29.8		
	45	78.9	39	68.4		
	2	3.5	1	1.8		
Her2/neu receptor					0.101	n.a.
• Negative • Positive • Missing	48	84.2	39	68.4		
	8	14.0	15	26.3		
	1	1.8	3	5.3		
Tumor stage [20]					0.001*	0.024*
• T1 • T2 • T3 • T4	16	28.1	28	49.1		
	26	45.6	28	49.1		
	10	17.5	1	1.8		
	5	8.8	0	0.0		
Nodal status [20]					0.036*	0.510
• N0 • N1 • N2 • N3	13	22.8	26	45.6		
	21	36.8	21	36.8		
	13	22.8	8	14.0		
	10	17.5	2	3.5		
Distant metastasis [20]					0.002*	n.a.
• cM0 • cM1	48	84.2	57	100.0		
	9	15.8	0	0.0		

The cases with ovarian metastases were matched on the time interval between the diagnosis of breast cancer and oophorectomy to an equally large cohort of controls without ovarian metastases, as shown in the flow chart of Fig 1. Univariate and multivariate logistic regression analyses were used to compare the cases and matched controls for the clinicopathological characteristics as indicated in the table. *P*-values < 0.05 were considered statistically significant.

* Values are statistically significant; n.a. = not applicable.

### Validation of the control population

Table 3 shows that, apart from the fact that more hormone-sensitive breast tumors were diagnosed in the matched controls than in the CCCN controls, 75.4% compared to 31.2%, respectively (*p* = 0.047), no statistically significant differences were found. These data indicate that the clinicopathological characteristics of the matched controls broadly corresponded to those of women whose ovaries remained *in situ*.

**Table 3:** Clinicopathological characteristics of patients diagnosed with primary invasive breast cancer at age < 41 years with and without oophorectomy.

Characteristics	Matched controls		CCCN controls		*p*-value
	N = 57	%	N = 4944	%	
Age at diagnosis of breast cancer, years—median (range)	36	(27–40)	37	(18–40)	0.347
Breast tumor localization					0.735
• Left • Right • Both • Missing	27	47.4	2552	51.6	
	30	52.6	2376	48.1	
	0	0.0	15	.3	
	0	0.0	1	0.0	
Histological subtype					0.303
• Ductal • Lobular • Ductolobular • Other	52	91.2	4391	88.8	
	4	7.0	209	4.2	
	1	1.8	143	2.9	
	0	0.0	201	4.1	
Scarff-Bloom-Richardson grade					0.741
• I • II • III • Missing	5	8.8	417	8.4	
	20	35.1	1241	25.1	
	31	54.4	2436	49.3	
	1	1.8	850	17.2	
Estrogen receptor					0.047*
• Negative • Positive • Missing	14	24.6	1117	22.6	
	43	75.4	1543	31.2	
	0	0.0	2284	46.2	
Progesterone receptor					0.070
• Negative • Positive • Missing	17	29.8	1303	26.4	
	39	68.4	1301	26.3	
	1	1.8	2340	47.3	
Her2/neu receptor					0.809
• Negative • Positive • Missing	39	68.4	1913	38.7	
	15	26.3	733	14.8	
	3	5.3	2298	46.5	
Tumor stage [20]					0.124
• T1 • T2 • T3 • T4 • Missing	28	49.1	2319	46.9	
	28	49.1	1989	40.2	
	1	1.8	360	7.3	
	0	0.0	197	4.0	
	0	0.0	79	1.6	
Nodal stage [20]					1.000
• pN0 • pN1 • pN2 • pN3 • Missing	26	45.6	2416	48.9	
	21	36.8	1665	33.7	
	8	14.0	560	11.3	
	2	3.5	248	5.0	
	0	0.0	55	1.1	
Distant metastasis [20]					0.100
• cM0 • cM1	57	100.0	4720	95.5	
	0	0.0	224	4.5	

Patients without ovarian metastases were indicated as matched controls, of whom selection is shown in the flow chart of Fig 1. Patients without oophorectomy were indicated as CCCN controls, of whom selection is illustrated in the flow chart of Fig 2. Univariate logistic regression analyses were used to compare the matched controls and CCCN controls for the clinicopathological characteristics as indicated in the table. *P*-values < 0.05 were considered statistically significant.

* Values are statistically significant.

## Discussion

In the current Dutch nationwide retrospective cohort study, we found that ovarian metastases occurred in 2.4% of young women diagnosed with primary invasive breast cancer who subsequently underwent an oophorectomy. This percentage is much lower than the previously reported prevalence rates of 13–47% [8–10]. The discrepancy between our findings and those reported in the literature can be explained by the fact that the prevalence rates were explored in different patient populations. In previous studies, the prevalence rates were primarily derived from clinical studies in patients with disseminated breast cancer who underwent therapeutic oophorectomy, and autopsy reports of patients who died of metastastic breast cancer [8–10]. Our findings were based on a nationwide cohort mainly consisting of young breast cancer patients in whom the ovaries were either removed prophylactically because of a positive family history and/or the presence of a *BRCA* gene mutation, or therapeutically because of hormone-sensitive breast cancer. Hence, our findings provide more insight into the prevalence of ovarian metastases in the general population of young breast cancer patients. Nonetheless, some remarks on the establishment of this prevalence rate should be made. Firstly, the prevalence of ovarian metastases was solely substantiated among young breast cancer patients who underwent an oophorectomy. The reason for this was that ovarian metastases can only be diagnosed with certainty by microscopic examination [21]. The prevalence of ovarian metastases among young breast cancer patients whose ovaries remained *in situ* thus remains elusive. This point might also be considered as a strength of the current study, as our findings are exclusively based on a large cohort of young breast cancer patients in whom the presence of ovarian metastases could be determined. Secondly, it should be noted that the majority of the ovarian tissues were not completely examined, since sequentially cut tissue sections were often not obtained using standard pathology procedures. As a result, malignant cells might have been overlooked, thereby potentially resulting in an underestimation of the prevalence of ovarian metastases among young breast cancer patients. Thirdly, the time between breast cancer and the onset of ovarian metastases was on average 42 months, whereas in patients who undergo ovarian tissue cryopreservation an oophorectomy is usually performed soon after initial diagnosis. Fourthly, the majority of the patients included in this study were treated with chemotherapy, which may have treated distant metastases if present. Lastly, 26% of the cases underwent an oophorectomy because their ovary appeared abnormal on ultrasound. Each of these factors might have affected the prevalence rate to some extent. Nonetheless, the prevalence rate based on the current study represents the closest possibility to come to a prevalence of ovarian metastases among young breast cancer patients who may undergo ovarian tissue autotransplantation, since frozen-thawed cortical ovarian fragments from patients who are willing to undergo ovarian tissue autotransplantation cannot be used to estimate the prevalence rate and examination of cortical ovarian tissue fragments from deceased patients will certainly yield too small study populations to draw reliable conclusions from.

The most striking difference between the cases and controls in the source population was the difference in time interval between the diagnosis of breast cancer and oophorectomy. Due to the retrospective study design, it was impossible to find out why the ovaries were much earlier removed in the controls than in the cases. Nevertheless, these findings suggest that the risk of developing ovarian metastases increases with the passage of time. Hence, in young breast cancer patients who wish to preserve their fertility, it seems important to perform an oophorectomy soon after the diagnosis of breast cancer in order to reduce the risk for the development of ovarian metastases. Besides, some recommendations can be proposed with respect to the site of ovarian tissue autotransplantation. As long as there is no accurate alternative to the current tumor detection approach available by which the actual ovarian autografts can be examined, it would be advisable to transplant the cortical ovarian fragments back to the remaining ovary rather than, for instance, a peritoneal window. After all, transplantation of the cortical ovarian fragments to the remaining ovary enables the complete removal of the grafted ovarian tissues at a later stage by simply extirpating the entire ovary, for instance when the patient’s family has been completed or when the ovarian grafts have ceased functioning. By contrast, in case the cortical ovarian fragments are transplanted to a peritoneal window, complete extraction of these fragments cannot be guaranteed, as it will be difficult to retrace the ovarian autografts within the peritoneum. Hence, transplantation to the remaining ovary should be preferred over transplantation to the peritoneum as it may further minimize the risk that tumor cells in the ovarian grafts ultimately develop into ovarian metastases. Lastly, in the patients who were diagnosed with ovarian metastases, it is plausible that tumor cells have disseminated very early after the onset of cancer and have long remained dormant before they formed overt metastases in the ovaries [22,23]. Our findings therefore do not alter the fact that minimal residual disease should be excluded in the actual ovarian autografts in order to avoid a cancer relapse following ovarian tissue autotransplantation.

Because the presence of ovarian metastases is inextricably linked to the time of oophorectomy, baseline risk factors could only be determined if the time interval between the cases and controls was comparable. The most suitable approach to achieve this would be to subject every young patient who is diagnosed with primary invasive breast cancer to a bilateral oophorectomy after a certain predefined time interval and subsequently evaluate whether ovarian metastases have developed. However, such an approach would obviously never be ethically acceptable. We therefore circumvented this by matching the 57 cases, of whom clinical data were available, to an equally large cohort of controls on this time interval, making accurate risk factor analyses possible. These risk factor analyses showed that a larger tumor size (i.e. > 5 cm) and the presence of inflammatory breast cancer resulted in an increased risk of developing ovarian metastases. Yet, because the matched controls did not fully reflect the general population of young breast cancer patients without ovarian metastases, the magnitude of association between the tumor stage and the risk of developing ovarian metastases has limited value for clinical practice.

Although other reports stated that lobular breast cancers are more likely to metastasize to the ovary than ductal breast cancers [21], we did not observe any significant differences in histological subtype between the cases and matched controls. This might be different in elderly women with breast cancer, as lobular breast cancers are more frequently diagnosed in older patients [24].

Information on *BRCA* gene mutation status was available from 10 cases (17.5%) and 21 matched controls (36.8%). Compiled data from 18 studies reporting a total of 1187 women with *BRCA* mutations who underwent risk-reducing salpingo-oophorectomy revealed only two patients (0.17%) with metastatic breast cancer in the ovaries [25]. Hence, the presence of a *BRCA* gene mutation does not seem to be associated with the risk of developing ovarian metastases in patients with breast cancer and was therefore not taken into account in our risk factor analyses. Nevertheless, risk-reducing salpingo-oophorectomy is often recommended to *BRCA* gene mutation carriers to reduce their risk of developing primary ovarian cancer [26].

As described above, patients were only enrolled in the current study if they had undergone ovarian surgery. Nevertheless, a comparison of our matched controls to young breast cancer patients whose ovaries remained *in situ* (CCCN controls) showed that the clinicopathological characteristics were broadly similar between the two groups. The reason that our matched controls were more often diagnosed with hormone-sensitive breast tumors relies on the fact that the indication for oophorectomy in these patients was primarily therapeutic. Hence, apart from the difference in hormone receptor expression, the intrinsic tumor characteristics of our matched controls were passably in line with those of young breast cancer patients whose ovaries remained *in situ*.

In conclusion, our research shows that secondary ovarian involvement is encountered in 2.4% of young breast cancer patients. In order to minimize the risk of developing ovarian metastases in young breast cancer patients who wish to preserve their fertility, we recommend early ovary removal followed by transplantation of cortical ovarian tissue fragments to the remaining ovary. Ultimately, when the patient’s family has been completed or when the ovarian grafts have ceased functioning, the remaining ovary to which the cortical ovarian tissue fragments were transplanted should preferably be removed in order to keep the risk of developing ovarian metastases as low as possible. In addition, we suggest a cautious approach to ovarian tissue autotransplantation in patients diagnosed with tumors > 5 cm and/or inflammatory breast cancer.

## Supporting Information

S1 Data Data of patients diagnosed with primary invasive breast cancer at age < 41 years with and without ovarian metastases. The cases with ovarian metastases were matched on the time interval between the diagnosis of breast cancer and oophorectomy to an equally large cohort of controls without ovarian metastases, as shown in the flow chart of Fig 1. N.A. = Not applicable; NED = No evidence of disease; AWD = Alive with disease; DOC = Dead of other cause; DOD = Dead of disease; DSS = Disease-specific survival.
(XLSX)

S2 Data Data of patients diagnosed with primary invasive breast cancer at age < 41 years who did not undergo an oophorectomy. This cohort of patients was compiled by the Comprehensive Cancer Center the Netherlands (CCCN) and indicated as CCCN controls in the study.
(XLSX)
